# MRI Visualization of *Staphyloccocus aureus*-Induced Infective Endocarditis in Mice

**DOI:** 10.1371/journal.pone.0107179

**Published:** 2014-09-17

**Authors:** Janine Ring, Verena Hoerr, Lorena Tuchscherr, Michael T. Kuhlmann, Bettina Löffler, Cornelius Faber

**Affiliations:** 1 Department of Clinical Radiology, University Hospital Münster, Münster, Germany; 2 Institute of Medical Microbiology, University Hospital Münster, Münster, Germany; 3 European Institute for Molecular Imaging, Westfalian Wilhelms-University, Münster, Germany; Karolinska Institutet, Sweden

## Abstract

Infective endocarditis (IE) is a severe and often fatal disease, lacking a fast and reliable diagnostic procedure. The purpose of this study was to establish a mouse model of *Staphylococcus aureus*-induced IE and to develop a MRI technology to characterize and diagnose IE. To establish the mouse model of hematogenous IE, aortic valve damage was induced by placing a permanent catheter into right carotid artery. 24 h after surgery, mice were injected intravenously with either iron particle-labeled or unlabeled *S. aureus* (strain 6850). To distinguish the effect of IE from mere tissue injury or recruited macrophages, subgroups of mice received sham surgery prior to infection (n = 17), received surgery without infection (n = 8), or obtained additionally injection of free iron particles to label macrophages (n = 17). Cardiac MRI was performed 48 h after surgery using a self-gated ultra-short echo time (UTE) sequence (TR/TE, 5/0.31 ms; in-plane/slice, 0.125/1 mm; duration, 12∶08 min) to obtain high-resolution, artifact-free cinematographic images of the valves. After MRI, valves were either homogenized and plated on blood agar plates for determination of bacterial titers, or sectioned and stained for histology. In the animal model, both severity of the disease and mortality increased with bacterial numbers. Infection with 10^5^ *S. aureus* bacteria reliably caused endocarditis with vegetations on the valves. Cinematographic UTE MRI visualised the aortic valve over the cardiac cycle and allowed for detection of bacterial vegetations, while mere tissue trauma or labeled macrophages were not detected. Iron labeling of *S. aureus* was not required for detection. MRI results were consistent with histology and microbial assessment. These data showed that *S. aureus*-induced IE in mice can be detected by MRI. The established mouse model allows for investigation of the pathophysiology of IE, testing of novel drugs and may serve for the development of a clinical diagnostic strategy.

## Introduction

Infective endocarditis (IE) is a chronic bacterial infection of the endocardium and the valves, often leading to severe or life-threatening conditions [1]. A frequent causative pathogen is *Staphylococcus aureus,* a Gram positive bacterium, often associated with an acute and destructive form of endocarditis that can cause destruction of valves and requires cadiovascular surgery. According to the Duke criteria [1], [2] diagnosis of IE is founded on two major indications: positive blood cultures and echocardiogram abnormalities, such as a pendulum-like intracardial mass or regurgitation jets. For definite diagnosis, repeated and combined transthoracic and transesophageal echocardiography have to be performed [1], [3]. However, negative findings occur regularly and the examination must be repeated [4]. Adequate therapy is often delayed, since the diagnostic latency averages longer than one month, which contributes to the high mortality rate of 20–40% [5], [6]. Therefore, a fast and reliable alternative diagnostic method is urgently required to improve treatment options. PET/CT has been shown to be able to identify IE, but to lack diagnostic reliability and is more suitable to detect infection of cardiovascular implantable electronic devices [7], [8]. Due to its excellent spatial resolution and tissue contrast MRI is a powerful tool for non-invasive diagnosis of disease, which is increasingly applied for examinations of the heart and cardiac function [9]. However, the low temporal resolution, compared to echocardiography, and the susceptibility to flow artifacts in the images, have rendered imaging of cardiac valves problematic. Although cardiac masses in the human heart are detectable by MRI, a specific MR diagnosis of bacteria-induced endocarditis is often not possible, since contrast-enhanced MR for detection of bacteria has not yet been developed. In experimental preclinical imaging approaches, optical and nuclear techniques have succeeded in imaging IE in mice [10]–[12]. However, MR has only been reported to be capable of imaging infiltration of immune cells in response to bacterial infections of other organs [13]–[19].

Two recent methodological advances may substantially advance MRI of endocarditis and allow for achieving highly sensitive MR detection of bacterial vegetations on the aortic valves. The unperturbed visualization of cardiac valves has become feasible with a novel self-gated cinematographic (CINE) ultra-short echo time (UTE) sequence [20]. This protocol allows for virtually complete suppression of flow artifacts and depiction of the valve motion over the full cardiac cycle in mice. Yet, the representation of valve thickening or pendulum-like masses in IE in mice has not been assessed previously. The second promising advance with potential impact on MR diagnosis of endocarditis in a mouse model was the implementation of a method to directly detect vegetations of labeled bacteria by MRI. *S. aureus* can be labeled with iron oxide nano particles in vitro and applied for induction of infections in mice. Vegetations in these models are readily observable in T2* weighted MRI, as previously shown in subcutaneous or semi-systemic infection models [21]. In the present work, we have developed a mouse model of *S. aureus*-induced IE and combined the CINE UTE MRI of the valves with iron-labeling of *S. aureus*, to assess whether MRI can detect IE.

## Materials and Methods

### Mouse model of *S. aureus*-induced infective endocarditis

The study involved male and female CD-1 mice weighing 35–50 g. Animals were euthanized either after the final MRI as described below or at defined humane endpoints (early signs of pain or distress, such as abnormal posture, lack of foraging behavior, dull and shaggy fur) by CO_2_ asphyxiation. Animals were inspected twice a day and received medication against post-surgical pain (Carprofen; Rimadyl, Pfizer animal health, NY, USA). All in vivo experiments were performed according to the guidelines of the European Regulations for Animal Welfare. The protocol was approved by the Landesamt für Natur, Umwelt und Verbraucherschutz Nordrhein-Westfalen (LANUV) (ID 87–51.04.2011.A003). The design of our study involved several groups of animals that received different treatment (see Figure S1, showing all groups of animals used in this study) to distinguish the effects of IE from the effects of surgery alone, of septic infection, and of infiltrating macrophages. It was further tested if labeling of bacteria was required for detection or provided better results as compared to infection with unlabeled bacteria.

**Figure 1:**
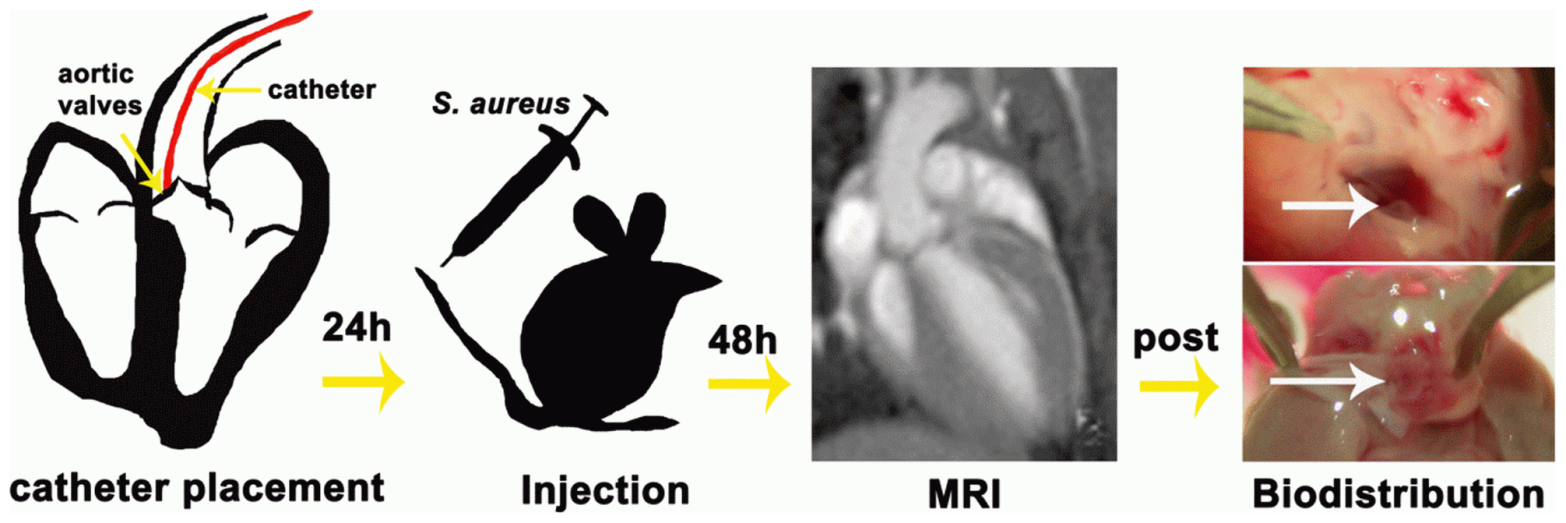
Schematic diagram of the endocarditis model. The experimental procedure involved four stages. First, valve trauma was induced by placing a 32-G catheter (red) at the aortic root via the right carotid artery. Second, *S. aureus* bacteria were injected via the tail vain 24 h after surgery. Third, MRI was performed 48 h after surgery. Fourth, animals were euthanized after MRI, hearts and organs were removed, inspected macroscopically and processed for microbial assessment or histology. Visual inspection showed clear, translucent valves (white arrow) for non-infected hearts (top) and yellow, thickened valves (white arrow) for colonized hearts (bottom).

For induction of IE in mice, we followed a procedure previously described by Gibson et al. [22], which involved surgical injury of the valve and, 24 h later, intravenous injection of bacteria (Fig. 1). In brief, endovascular *S. aureus* infection was facilitated by irritating the aortic valves using a 32-G polyurethane catheter (18 mm of the tubing was cut and heat-sealed at both ends) permanently placed at the aortic root via the right carotid artery. This position of the distal end of the catheter was reliably reached by advancing the catheter slowly until the vibrating motion from the fast moving leaflets was encountered. During surgery mice were anesthetized with 2% isoflurane and received medication against post-surgical pain (Carprofen; Rimadyl, Pfizer animal health, NY, USA). In sham-surgery animals, to assess the effect of infection without formation of IE, the catheter was removed immediately after placement.

In an initial group of n = 33 mice, the optimum concentration of bacteria for stable induction of IE was tested. Three different intravenous bacterial concentrations, 10^4^ (n = 8), 10^5^ (n = 18), and 10^6^ (n = 7) colony forming units (CFU) of *S. aureus* were used and administered in 100 µl PBS via the tail vein 24 h after catheter placement. All following experiments were performed either with 10^5^ CFU (n = 49) inoculation 24 h after surgery, or with sham-infection groups that did not receive bacteria (n = 8), to assess the effect of surgery alone. Among the mice that obtained 10^5^ CFU, n = 18 obtained unlabeled bacteria and n = 31 bacteria labeled with iron oxide nano particles (VSOP C200, Ferropharm Teltow, Germany), to assess whether labeling of bacteria is required for detection. A subset of animals of both groups (n = 4 and n = 9, infected with labeled and unlabeled bacteria, respectively) additionally received intraveneously 13 µl of a VSOP suspension (0.5 mM Fe/ml) 2 h and 6 h after infection. These particles are partly taken up by macrophages that are recruited by the immune response [14], [15], [18], [23], and allow separating signal originating from the immune response from contrast changes due to bacteria. Another subset of both groups (n = 8 and n = 3, labeled and unlabeled bacteria) had received sham surgery (catheter removed immediately). For an overview see Figure S1, showing all groups of animals used in this study.

### Bacterial strain and labeling with iron oxide nano particles

For all infections the methicillin-susceptible and highly virulent wildtype *S. aureus* strain 6850 (ATCC 53657) [24] was used. Cell concentration was determined spectroscopically by measuring the OD578 (optical density at 578 nm) in combination with counting CFU on blood agar after incubation overnight. For labeling with iron oxide a cell suspension of 10^9^ CFU/ml of *S. aureus* cells was incubated with rhodamine labeled 5-nm VSOP C200 at a concentration of 19.2 µM as described previously [21].

### MR Imaging

MRI was performed 48 h after catheter placement at the site of aortic valves. Imaging was performed on a Bruker BioSpec 94/20 equipped with a 1 T/m gradient system and a 35-mm volume coil. During MRI, the animals were anesthetized with 1–2% Isoflurane and were monitored for respiration and heart rate. Positioning of the image slice was performed using a self-gated CINE FLASH sequence (TR/TE: 6/3 ms, FA: 30°, FOV: (3.20 cm)^2^, MTX: 256^2^). For visualization of the infected aortic valves over the cardiac cycle a self-gated CINE UTE sequence was used (TR/TE: 5/0.31 ms, FA: 15°, FOV: (3.20 cm^2^), MTX: 256×256, slice: 1 mm, scan duration: 12∶08 min). 20 frames per cardiac cycle were reconstructed, exploiting the retrospective gating method. A total of n = 59 mice was investigated by MRI: n = 3 with 10^6^ CFU (initial group), n = 6 with 10^4^ CFU (initial group), n = 42 with 10^5^ CFU (among these 2 from the initial group), and n = 8 with sham-infection. For an overview see Figure S1, showing all groups of animals used in this study. All CINE MR images (long-axis view) were scored independently by two blinded readers, with more than ten and nine years of experience in mouse MRI, respectively. A six-level endocarditis score was used, which included the criteria valve thickening, hypointesities on the leaflets or the aortic wall, and presence of pendulum-like intra-cardiac masses: 0 – no abnormalities in any criterion; 1 – uncertain abnormalities in one criterion; 2 – uncertain abnormalities in more than one criterion or small abnormalities in one criterion; 3 – small abnormalities in more than one criterion or clear abnormality in one criterion; 4 – pronounced abnormality in one criterion or clear abnormality in all criteria; 5 – large signal change in large area. If additionally a disturbed motion of the leaflets or an abnormal flow pattern (structured flow artefacts) was observed, the score was raised by one level. In addition to the endocarditis score the criterion pendulum-like intra-cardiac masses was rated separately from 0 – no cardiac mass, to 5 – large mass with additional hypointensity.

### *Ex vivo* analysis

After MRI, animals were euthanized by CO_2_ asphyxiation if used for microbial assessment or transcardial perfusion under deep anesthesia (4% isoflurane) if used for histology. Hearts were explanted and processed for further analysis. Following macroscopic evaluation of the valves under a binocular microscope they were dissected from the rest of the heart and either fixed in 4% formalin and embedded in paraffin for histological evaluation, or homogenized for microbial assessment. Due to this procedure and the fact that some mice reached the defined endpoints before obtaining MRI, numbers of total mice, MRI investigations and microbial analysis were different. According to the common procedure in infection biology and microbiology, bacterial counts of tissue homogenates plated on blood agar were used as gold standard for severity of the infection. Histology was performed only for additional visualization in few animals per group, since no validated method is available, to determine bacterial counts from histological slices.

### Microbiological evaluation and Histology

In addition to the aortic valves of n = 44 mice, the myocardium and kidneys were homogenized, serially diluted in PBS and plated on blood agar plates for determination of bacterial titers.

To verify that vegetations were actually caused by the injected strain 6850, homogenates of aortic valves of selected animals were directly used for pulsed field gel electrophoresis (PFGE, data not shown). DNA macrorestriction and separation of fragments by PFGE were conducted using standardized procedure [25]. DNA was digested with SmaI (New England Biolabs, Ipswich, MA, USA). PFGE conditions were 6 V/cm at 11.3°C for 23 hours, with pulses of 5 to 35 seconds. Electrophoresis was performed using a CHEF DR II instrument (Bio-Rad, Hercules, CA, USA). Band patterns were visually interpreted following the criteria of Tenover et al. [26].

For histological analysis paraffin-embedded valves were transversally cut in serial sections and Gram-positive bacteria were stained with crystal violet/iodine and Prussian blue according to standard protocols. To detect fibrin and inflammatory cells the hematoxylin and eosin stain was used. Macrophages were stained according to standard protocols using a monoclonal rat anti-mouse Mac3 antibody followed by incubation with biotin mouse anti-rat IgG1/2a (BD Bioscience, Heidelberg, Germany) as secondary antibody, Streptavidin/HRP (Dako, Hamburg, Germany) and DAB detection System (Vector Laboratories, Inc., CA, USA). Nuclei were counterstained with Mayer’s Hematoxylin Solution (Sigma Aldrich, Schnelldorf, Germany). Tissue sections were examined under the light microscope (Nikon TE 2000-S, Nikon-Düsseldorf, Germany) at 60× and 100× primary magnification.

### Statistical Analysis

Results are presented as mean ± standard error. Statistical analyses were performed using the GraphPad Software (GraphPad Software Inc., San Diego, USA). For bacterial titers, a one way ANOVA based on a Student’s t-test was performed, comparing columns pairwise after Bonferroni correction. For the MRI data, the two independent sets of scores were used and a Kruskal-Wallis test was performed for two times n (number of animals) data points in each group. A value of p<0.05 was considered to be significant.

## Results

**Figure 2:**
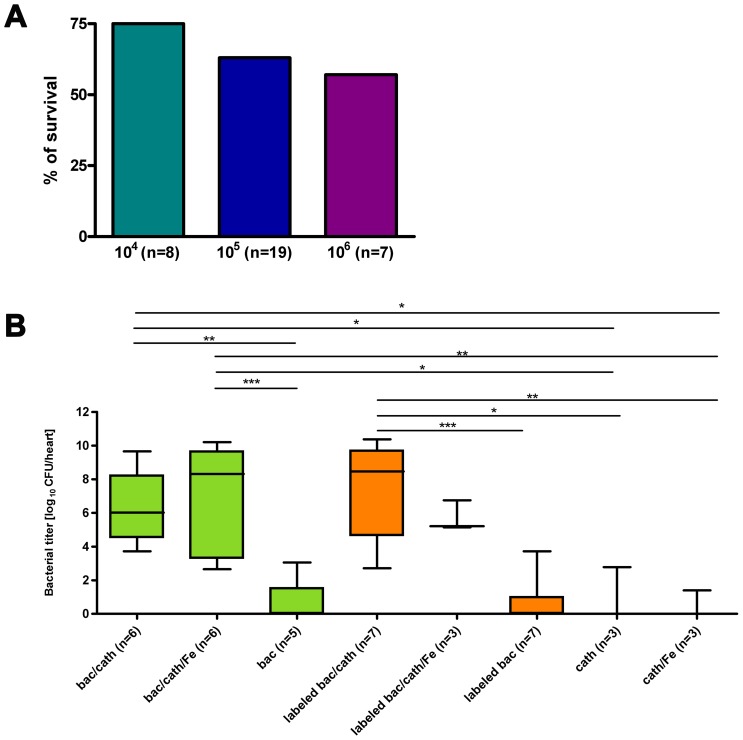
Feasibility of the endocarditis model. (A) Survival rate 24 h after infection with different concentrations of bacteria (10^4^, 10^5^ and 10^6^ CFU) for an initial group of mice. (B) Bacterial titer on the valves 24 h after infection with 10^5^ CFU for the different groups used in this study. Groups were: mice with permanent catheter and infection with unlabeled bacteria (bac/cath); permanent catheter, infection with unlabeled bacteria and additional VSOP administration (bac/cath/Fe); sham surgery and infection with unlabeled bacteria (bac); permanent catheter and infection with VSOP-labeled bacteria (labeled bac/cath); permanent catheter, infection with VSOP-labeled bacteria and additional VSOP administration (labeleld bac/cath/Fe); sham surgery and infection with labeled bacteria (labeled bac); permanent catheter only (cath); permanent catheter and additional VSOP administration (cath/Fe). Box plots show median, 25^th^ and 75^th^ percentiles and extreme values. Significant differences are indicated: * p<0.05, ** p<0.01, *** p<0.001.

IE was reliably induced by the surgical procedure involving placement of a catheter at the aortic valve and subsequent i.v. application of *S. aureus* (Fig. 1). Survival rate after injection of *S. aureus* was assessed in the initial group of n = 33 mice. It was dependent on bacterial number in the inoculum, 75% for 10^4^ CFU (6/8), and 66% for 10^5^ CFU (12/18) and 57% for 10^6^ CFU (4/7) one day after infection (Fig. 2a). All three concentrations of bacteria in the administered inoculum, reliably caused infection of the valves with high numbers of CFU (10.61±0.56 for 10^6^ (n = 2); 5.71±0.99 for 10^5^ (n = 3); 5.29±3.64 for 10^4^ (n = 2), all as log10). Due to the very high number of CFU and the occurrence of severe sepsis in some mice infected with 10^6^ CFU, this concentration was not continued after the initial infections. Figure 2b summarizes the bacterial titers found for all mice infected with 10^5^ CFU and subjected to microbial assessment. All four groups of infected mice with permanent catheter showed significantly higher titers compared to sham-surgery or sham-infection groups. Bacterial numbers on the valves of mice infected with unlabeled bacteria (6.25±0.82, n = 6) were not significantly different for mice infected with labeled bacteria (7.23±1.16, n = 7). Also no differences were found between mice that had obtained injection of VSOP after infection, suggesting that iron particles do not inhibit bacterial growth (7.09±1.25 (n = 6) for unlabeled bacteria and 5.70±0.53 (n = 3) for labeled bacteria. In contrast, permanent placement of the catheter was found crucial for the development of bacterial vegetations. In animals with sham surgery (catheter immediately removed after placement), bacteria were found on the valves in only 1 of 5 animals and 2 of 7 animals for infection with unlabeled and labeled bacteria, respectively, resulting in average bacterial numbers of 0.61±0.61 (n = 5) and 0.67±0.53, (n = 7), respectively (Fig. 2b). In the two sham-infection groups with permanent catheter only (no infection, one group with additional VSOP injection) low numbers of bacteria were found in 1 of 3 animals each (0.93±0.93 CFU (n = 3) and 0.47±0.47 CFU (n = 3) without and with VSOP injection, respectively, Fig. 2b). Bacterial counts in the kidney showed that mice did not suffer from a generalized sepsis. No significant difference in bacterial numbers were observed between animals with and without catheter for unlabeled and labeled bacteria (3.04±1.27 vs 0.82±0.51, p = 0.17 and 4.92±1.34 vs 2.96±0.76, p = 0.23, respectively, see Figure S2, Table S2).

**Figure 3:**
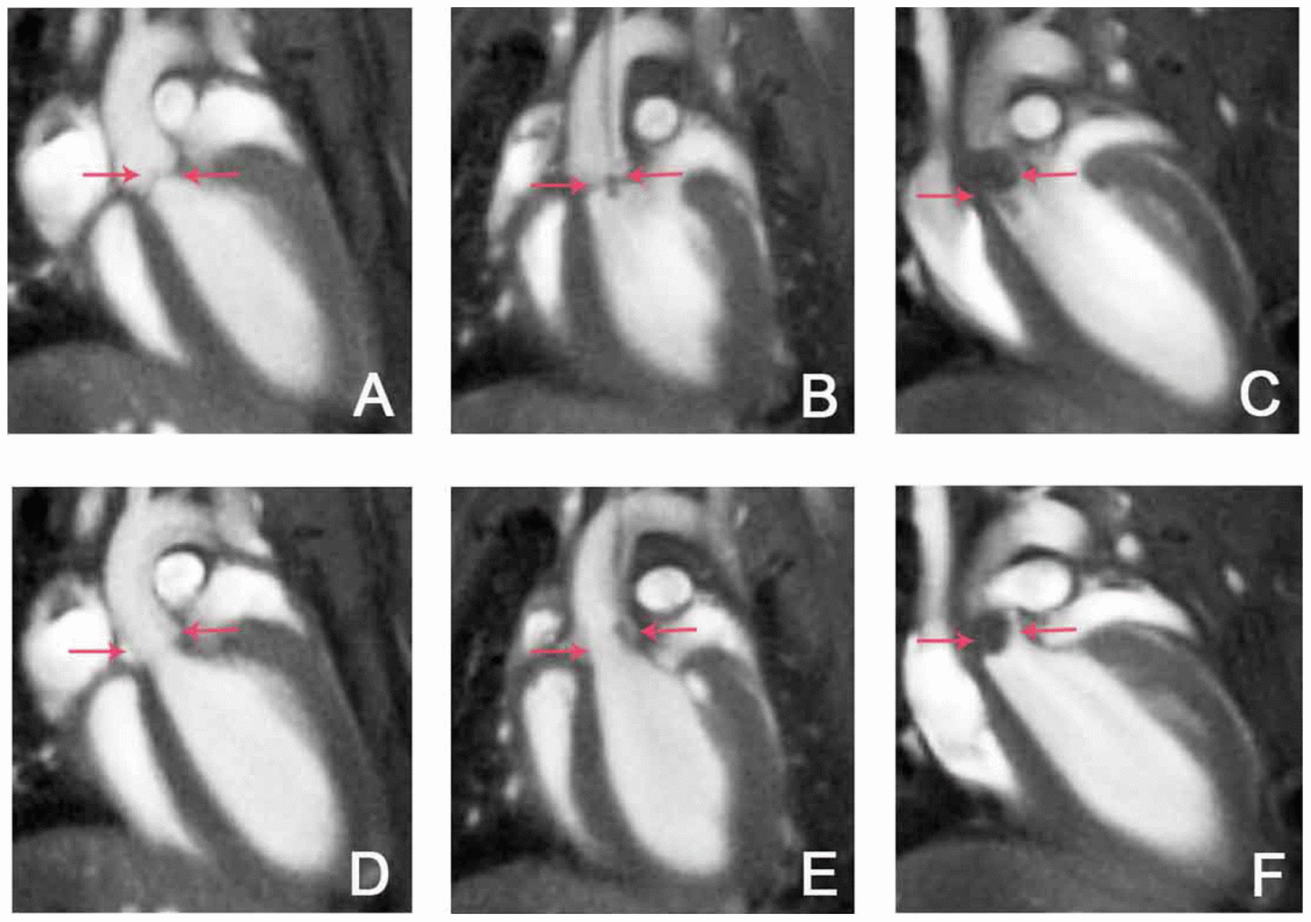
MRI detection of *S. aureus* vegetations on the aortic valve. Exemplary long axis views of the mouse heart acquired with self-gated CINE UTE MRI (TR/TE : 5/0.31 ms, FA: 15°, resol: (125 µm)^2^, MTX: 256×256, slice thickness: 1 mm, scan duration: 12∶08 min) show the aortic valves in closed (A–C) and open (D–F) state. Flow artifacts were almost completely suppressed by the use of self-gated UTE MRI. (A,D) Images of a mouse with sham surgery infected with unlabeled bacteria show normal valves (arrows). (B, E) In images of a catheterized mouse infected with unlabeled bacteria the catheter as well as valve thickening and an additional intracardial mass is visible (arrows). (C, F) Large hypointensities on the valves (arrows) are observed in this catheterized mouse infected with iron labeled bacteria.

**Figure 4:**
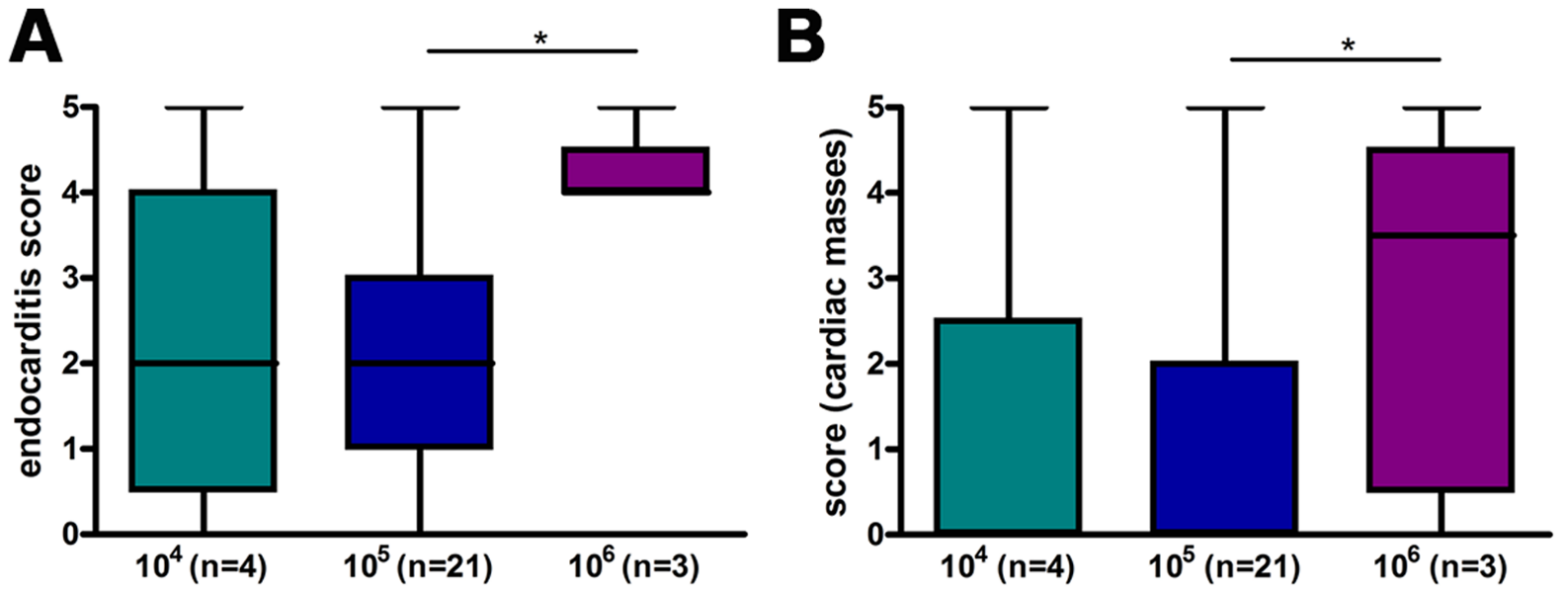
MRI scores depend on number of infecting bacteria. Endocarditis score (A) and score for presence of intra-cardiac masses (B) for the initial group of mice infected with different concentrations of bacteria. MRI was performed 24 h after infection with *S. aureus* and scored on a six-level scale from 0 (inconspicuous) to 5 (most conspicuous). n represents the number of animals. As two independent scorings were used the number of data points per column is 2×n. Box plots show median, 25^th^ and 75^th^ percentiles and extreme values. Significant differences are indicated: * p<0.05.

MRI using self-gated CINE UTE yielded high resolution images of the valves over the full cardiac cycle. The catheter was visible in the respective image slices, but no artifacts from the catheter were observed. Exemplary images are shown in Figure 3, illustrating diverse manifestations of aortic valve thickening, hypointensities on the leaflets, intra-cardiac masses, or the catheter. Abnormalities were exclusively observed in tissue irritated by the catheter: at the aortic root, the aortic valve and the myocardium. Large hypointensities (Fig. 3c) on the aortic valves were observed for some infected mice. However, these occurred not only for iron-labeled bacteria, but also for unlabeled bacteria. All images were rated independently by two blinded readers using the endocarditis score as described in the methods section. Scores did not show significant differences between 10^4^ and 10^5^ CFU in the inoculum (2.25±0.73 and 2.21±0.25) but were significantly increased for 10^6^ CFU (4.17±0.17) (Fig. 4 a). Likewise, scores for the additional criterion presence of intra-cardiac masses were only significantly increased for 10^6^ CFU (1.25±0.73 and 0.95±0.20 versus 2.84±0.79, respectively) (Fig. 4 b).

**Figure 5:**
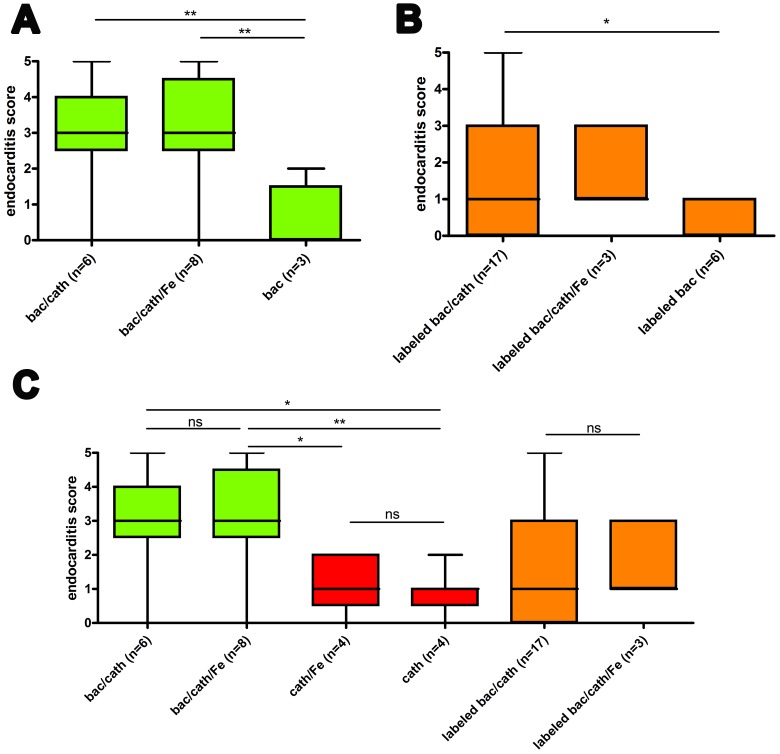
MRI detects infective endocarditis. Endocarditis scores (24 h after infection with 10^5^ CFU *S. aureus*) for the groups defined in Fig. 2. (A) Scores for mice infected with unlabeled bacteria versus sham-surgery group. (B) Scores for mice infected with labeled bacteria versus sham-surgery group. (C) Scores for infected mice (both labeled and unlabeled) versus sham-infection groups, and score for mice with versus without injection of VSOP. n represents the number of animals. As two independent sets of scores were used the number of data points per column is 2×n. Box plots show median, 25^th^ and 75^th^ percentiles and extreme values. Significant differences are indicated: * p<0.05, ** p<0.01, ns not significant.

The endocarditis scores are summarized in Fig. 5 for six groups of mice that were infected with 10^5^ CFU and two sham-infection groups (with permanent catheter placement but no infection). Scores for mice with permanent catheter and infection with unlabeled bacteria showed significantly higher scores compared to sham-surgery and sham-infection groups (Fig. 5a,c). See also Table S3 and Movies S1–S6. The lower score of the sham-surgery group confirmed that tissue injury is required for formation of endocarditis and the lower score of the uninfected group provided evidence that indeed bacterial vegetations and not the wound was detected. In opposite to the expectations, for mice with permanent catheter and infection with labeled bacteria such significant differences to the sham groups were not found (Fig. 5b), although the scores for iron-labeled compared to unlabeled bacteria were not significantly different (Fig. 5c). Further, there was no significant difference between groups with and without additional injection of iron oxide particles for both infected and not-infected mice (Fig. 5c). This observation strongly supports the notion that vegetations of *S. aureus* were observed on the valves and not recruited macrophages.

**Figure 6:**
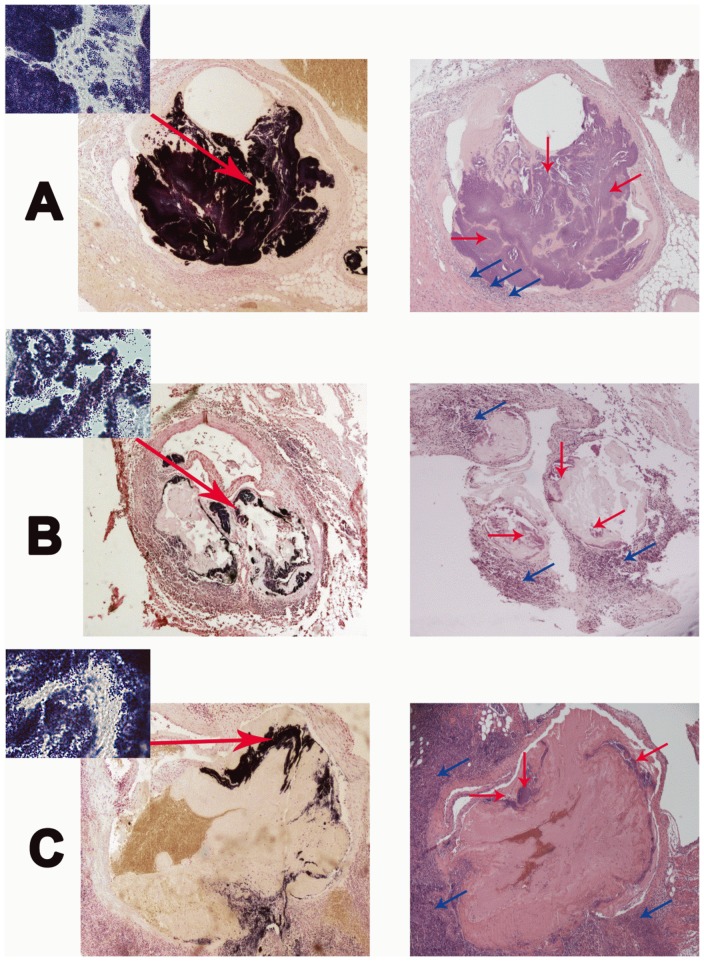
Histology of aortic valves. Gram staining (left column) revealed large colonies of Gram-positive bacteria on the valves in mice infected with (A) unlabeled bacteria, (B) labeled bacteria, and (C) labeled bacteria and additional administration of VSOP to label macrophages. Large arrows indicate where 100-fold magnifications were taken. Hematoxylin-esoin staining (right column) confirmed the presence of bacteria (red arrows), displayed neutrophil recruitment (blue arrows) and showed valve thickening due to early deposition of connective tissue, but did not show large numbers of infiltrating immune cells. Magnification, 4 fold (inset 100 fold).

Following the MRI exam, mice were euthanized and hearts explanted. Macroscopic images showed thickened valves of infected mice, while in the control mice valves appeared translucent without signs of bacterial colonization (see Fig. 1). Histological analysis revealed large vegetations of coccal-shaped Gram-positive bacteria on the valves of infected mice (Fig. 6). *S. aureus* strain 6850 was confirmed by PFGE of valve homogenates. Hematoxylin-eosin staining confirmed the presence of bacterial colonies, but did not give evidence for massive immune response by large numbers of infiltrating macrophages (Fig. 6), which was further corroborated by Mac3 staining (data not shown). Prussian blue staining of the valves was negative throughout, indicating the absence of substantial amounts of iron in the vegetations. In summary, macroscopic and microscopic imaging of explanted hearts was consistent with the MRI observations and supports the notion that bacteria were directly detected by MRI and not the iron label or infiltrating immune cells.

## Discussion

We have established a mouse model of *S. aureus*-induced IE. Formation of bacterial vegetations on the valves can be detected by MRI using self-gated CINE UTE MRI. Our data give evidence that the direct effects of the local bacterial infection are observed rather than the immune response. Neither tissue injury alone, induced by placement of a permanent catheter without infection, nor labeling of macrophages was detected in MRI. However, in contrast to our previous observation that iron labeling is required to detect *S. aureus* in animal models of infection [21], endocarditis was also detectable with unlabeled bacteria. Bacterial numbers after infection with labeled and unlabeled bacteria were not significantly different, confirming previous observations that iron-labeling had no adverse effect on growth behavior [21]. The lack of an effect of iron on image contrast is most likely due to the fast growth of the vegetation. Assuming that only a fraction of the 10^5^ injected bacteria contributed to initial formation of the vegetation, and considering that more than 10^6^ bacteria were found on the valves, iron has been diluted by several orders of magnitude. Such strong dilution is supported by the lack of detectable iron in Prussian blue stains. The strong hypointensities that were observed on the valves of some mice can most likely be attributed to blood clots, resulting from a combination of tissue injury and infection. This notion is in line with previous observations that valve colonization by *S. aureus* triggers blood clotting [27]. However, to finally prove that blood clots are the origin would require a detailed one-to-one correlation with histology, since other MRI techniques such as relaxation time mapping are not available for tiny fast moving structures as the aortic valves. Our observation that tissue injury is required for formation of bacterial vegetations on the valves supports previous studies that have shown a pivotal but ambiguous role of adhesins in formation of endocarditis [28]–[31].

In this study, we have compared the MRI results to counts of bacterial numbers from tissue homogenates, which is the established gold standard in infection biology and microbiology. Due to variations in bacterial numbers and severity of the infection, which are inherent to the infection process in the mouse model, only group averages can be compared. A quantitative correlation of MRI with histology is not possible, because there is no microbiological method established to reliably quantify the extent of vegetations and bacterial numbers from histological slices.

Our mouse model and MRI protocol have immediate impact on preclinical research since this approach now allows for addressing issues like the role of individual virulence factors such as adhesins or toxins in *S. aureus*-induced IE. The precise imaging of the time course of the infection will provide detailed insight into pathomechanisms and give access to bacterial virulence factors, infection dynamics, or pathogen-host interactions. Further, our mouse model is ideally suited to test novel drugs or treatment regimens. The site of infection is directly exposed to the blood stream, which simplifies pharmacokinetics for initial in vivo affinity tests of novel drug candidates.

For a potential clinical application, our study may serve as a paradigm that shows the feasibility to detect bacterial vegetations on cardiac valves by MRI. In endocarditis patients, the predictive value of MRI is most likely higher than in the mouse model. Lower heart rates, slower motion of the leaflets, and lower magnetic field strength may allow for improved image quality. Further, the lack of artificial tissue irritation excludes one potential confounder. Therefore, the particular finding that iron-labeling is not required, suggests that MRI holds promise as a tool for the diagnosis of endocarditis. For pathogen-specific and reliable diagnosis, however, a combination with a molecular marker will be required. A future diagnostic strategy may use targeted iron oxide nano particles that could be administered to the patient and label the pathogen on the valve. Pathogen-specific adhesins might be a feasible structure to be targeted. Thus, a tracer-based MR investigation may provide fast and reliable diagnosis of IE, helping to greatly improve treatment options and outcome.

## Conclusions

We have established a mouse model of *S. aureus*- induced IE that can be observed by MRI using self-gated CINE UTE, and may be used for the longitudinal monitoring of disease progression. Thus, it may constitute a paradigm for investigations of bacterial pathophysiology, testing of novel drugs, and development of a clinical diagnostic strategy.

## Supporting Information

Figure S1 **Study Design.** Schematic of different groups of animals used in this study.
(TIF)

Figure S2 **Box plot of bacterial titers in kidney 24 h after infection.**
(PDF)

Table S1 **Bacterial titers on the heart valves of individual animals.**
(PDF)

Table S2 **Bacterial titers in the kidneys of individual animals.**
(PDF)

Table S3 **Endocarditis scores for individual animals.** Exemplary data sets that are available as movies are indicated in the last column.
(PDF)

Checklist S1 **ARRIVE Guidelines checklist.**
(PDF)

Movie S1 **Exemplary CINE MRI for endocarditis score 0.**
(GIF)

Movie S2 **Exemplary CINE MRI for endocarditis score 1.**
(GIF)

Movie S3 **Exemplary CINE MRI for endocarditis score 2.**
(GIF)

Movie S4 **Exemplary CINE MRI for endocarditis score 3.**
(GIF)

Movie S5 **Exemplary CINE MRI for endocarditis score 4.**
(GIF)

Movie S6 **Exemplary CINE MRI for endocarditis score 5.**
(GIF)
