# Supplementary material for: MRI Visualization of Staphyloccocus aureus-Induced Infective Endocarditis in Mice
Source: PLoS One. 2014 Sep 17;9(9):e107179. doi: 10.1371/journal.pone.0107179 (PMC4167704; doi:10.1371/journal.pone.0107179)
Supplement: Figure S2 — Box plot of bacterial titers in kidney 24 h after infection. (PDF) [file pone.0107179.s002.pdf]

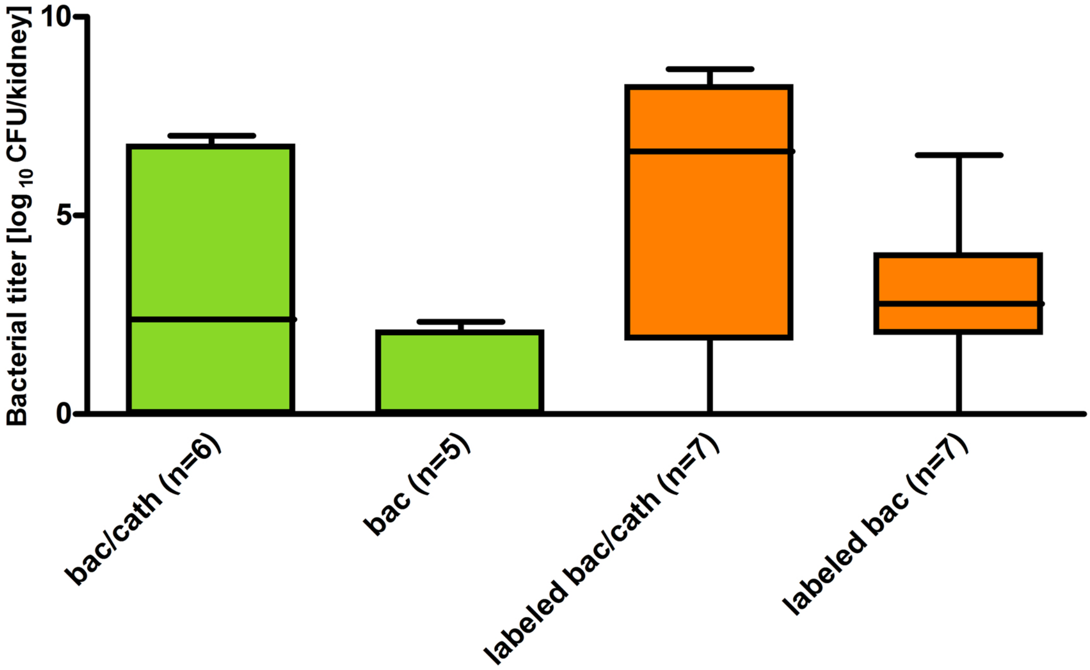

Suppl. Fig. 2: **Infection does not cause generalized sepsis**. Bacterial titer in the kidneys 24 h after infection with 10^5 CFU for the different groups used in this study. Groups were: mice with permanent catheter and infection with unlabeled bacteria (bac/cath); sham surgery and infection with unlabeled bacteria (bac); permanent catheter and infection with VSOP-labeled bacteria (labeled bac/cath); sham surgery and infection with labeled bacteria (labeled bac). Box plots show median, 25th and 75th percentiles and extreme values. No significant differences were observed.
