# Supplementary material for: MRI Visualization of Staphyloccocus aureus-Induced Infective Endocarditis in Mice
Source: PLoS One. 2014 Sep 17;9(9):e107179. doi: 10.1371/journal.pone.0107179 (PMC4167704; doi:10.1371/journal.pone.0107179)
Supplement: Table S1 — Bacterial titers on the heart valves of individual animals. (PDF) [file pone.0107179.s003.pdf]

Group A (bac/cath)	Bacterial titer [log_{10}CFU/heart valve]
1	6,64
2	3,72
3	6,76
4	5,39
5	5,33
6	9,66
**Group B (bac/cath/Fe)**	
1	7,77
2	2,66
3	3,92
4	10,21
5	9,09
6	8,87
**Group C (bac)**	
1	0,00
2	0,00
3	0,00
4	0,00
5	3,06
**Group D (labeled bac/cath)**	
1	8,47
2	2,72
3	4,64
4	5,70
5	9,70
6	10,37
7	9,48
**Group E (labeled bac/cath/Fe)**	
1	5,21
2	5,14
3	6,75
**Group F (labeled bac)**	
1	0,00
2	0,00
3	0,00
4	0,00
5	3,72
6	1,00
7	0,00
**Group G (cath)**	
1	2,78
2	0,00
3	0
**Group H (cath/Fe)**	
1	0,00
2	0
3	1,40
