# Supplementary material for: MRI Visualization of Staphyloccocus aureus-Induced Infective Endocarditis in Mice
Source: PLoS One. 2014 Sep 17;9(9):e107179. doi: 10.1371/journal.pone.0107179 (PMC4167704; doi:10.1371/journal.pone.0107179)
Supplement: Table S2 — Bacterial titers in the kidneys of individual animals. (PDF) [file pone.0107179.s004.pdf]

Group A (bac/cath)	Bacterial titer [log_{10}CFU/kidney]
1	6,46
2	7,00
3	0,00
4	0,00
5	3,38
6	1,40
**Group C (bac)**	
1	0,00
2	0,00
3	0,00
4	2,32
5	1,78
**Group D (labeled bac/cath)**	
1	7,16
2	8,23
3	6,61
4	8,69
5	0,00
6	1,88
7	1,88
**Group F (labeled bac)**	
1	6,52
2	4,00
3	3,18
4	0,00
5	2,27
6	2,78
7	2,01
