# Supplementary material for: MRI Visualization of Staphyloccocus aureus-Induced Infective Endocarditis in Mice
Source: PLoS One. 2014 Sep 17;9(9):e107179. doi: 10.1371/journal.pone.0107179 (PMC4167704; doi:10.1371/journal.pone.0107179)
Supplement: Table S3 — Endocarditis scores for individual animals. Exemplary data sets that are available as movies are indicated in the last column. (PDF) [file pone.0107179.s005.pdf]

	Endocarditis score		Endocarditis score
**Group A (bac/cath)**	first reader	second reader	Cine (example of score 0-5)
1	3	3	
2	3	3	
3	2	3	
4	5	5	x (5)
5	4	4	x (4)
6	0	0	
**Group B (bac/cath/Fe)**			
1	1	0	
2	4	4	
3	5	5	
4	5	5	
5	0	3	
6	4	3	
7	3	2	
8	3	3	
**Group C (bac)**			
1	0	0	
2	0	0	
3	2	1	
**Group D (labeled bac/cath)**			
1	0	0	
2	3	2	
3	5	4	
4	4	3	
5	2	3	
6	1	2	
7	1	1	
8	0	0	x (0)
9	1	1	
10	4	5	
11	3	3	x (3)
12	0	1	
13	0	2	
14	3	2	
15	1	0	
16	0	0	
17	1	1	
**Group E (labeled bac/cath/Fe)**			
1	3	3	
2	1	1	
3	1	1	
**Group F (labeled bac)**			
1	1	1	
2	0	0	
3	0	1	
4	1	1	
5	0	0	

6	0	0	

Group G (cath)

1	0	1	
2	1	0	
3	1	2	
4	1	1	x (1)

Group H (cath/Fe)

1	0	1	
2	1	2	
3	2	2	x (2)
4	1	0	
