# Supplementary figures and images for: MRI Visualization of Staphyloccocus aureus-Induced Infective Endocarditis in Mice
Source: PLoS One. 2014 Sep 17;9(9):e107179. doi: 10.1371/journal.pone.0107179 (PMC4167704; doi:10.1371/journal.pone.0107179)

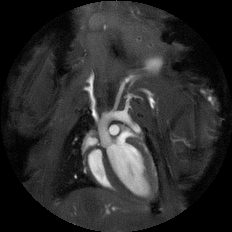

Supplement: Movie S1 — Exemplary CINE MRI for endocarditis score 0. (GIF) [file pone.0107179.s007.gif]

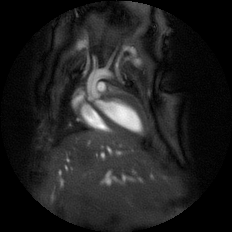

Supplement: Movie S2 — Exemplary CINE MRI for endocarditis score 1. (GIF) [file pone.0107179.s008.gif]

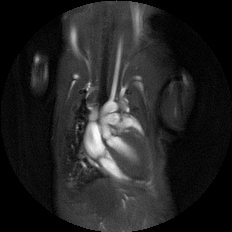

Supplement: Movie S3 — Exemplary CINE MRI for endocarditis score 2. (GIF) [file pone.0107179.s009.gif]

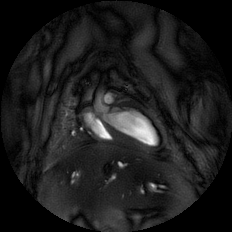

Supplement: Movie S4 — Exemplary CINE MRI for endocarditis score 3. (GIF) [file pone.0107179.s010.gif]

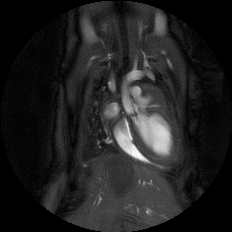

Supplement: Movie S5 — Exemplary CINE MRI for endocarditis score 4. (GIF) [file pone.0107179.s011.gif]

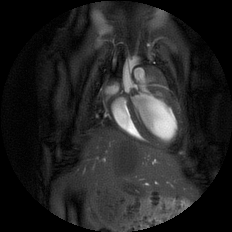

Supplement: Movie S6 — Exemplary CINE MRI for endocarditis score 5. (GIF) [file pone.0107179.s012.gif]
